# The Impact of Intraoperative Radiotherapy on Margin Positive Locally Advanced Rectal Cancer: A Propensity‐Matched Analysis of The National Cancer Database

**DOI:** 10.1002/jso.70102

**Published:** 2025-09-26

**Authors:** Metincan Erkaya, Salih Karahan, Mustafa Oruc, Sudha R. Amarnath, Jacob A. Miller, Ali Alipouriani, Brogan Catalano, Scott Steele, Emre Gorgun

**Affiliations:** ^1^ Department of Colorectal Surgery, Cleveland Clinic Digestive Disease and Surgery Institute Cleveland Ohio USA; ^2^ Department of Radiation Oncology Cleveland Clinic Taussig Cancer Institute Cleveland Ohio USA

**Keywords:** intraoperative radiotherapy, National Cancer Database, neoadjuvant, rectal cancer

## Abstract

**Purpose:**

Intraoperative radiotherapy (IORT) is utilized as an adjunctive treatment in advanced rectal cancer, particularly in cases with threatened surgical margins. Although IORT has shown benefits in enhancing local tumor control, its impact on overall survival (OS) remains unclear. This study assesses the effect of IORT on survival outcomes using a large cohort from the National Cancer Database (NCDB) and examines factors influencing its application in clinical practice across the United States.

**Methods:**

The National Cancer Database was retrospectively reviewed (2006–2019) to identify patients with pathological T3–T4, M0 rectal cancer who underwent surgery following neoadjuvant chemotherapy. Patients with microscopically residual margin‐positive were included and categorized into two groups: those who received neoadjuvant radiotherapy (RT) and those treated with intraoperative radiotherapy (IORT) combined with adjuvant/neoadjuvant RT. Groups were propensity score–matched (1:4) to balance baseline characteristics. The primary outcome was 5‐year overall survival (OS), assessed using Kaplan–Meier analysis and Cox proportional hazards modeling.

**Results:**

Among 1,788 patients with margin‐positive rectal cancer, IORT was administered to 119 patients (6.7%) while 1,669 patients (93.3%) received neoadjuvant RT. Patients receiving IORT were younger, more likely to have private insurance, more frequently treated at academic/research programs, and more commonly underwent pelvic exenteration and Multiagent chemotherapy. After propensity score matching, 119 IORT patients were compared with 476 neoadjuvant RT patients. IORT was associated with lower mortality in univariate analysis (HR: 0.63; *p* < 0.001); however, this benefit was attenuated after adjusting for confounders (HR: 0.84; *p* = 0.07). The 5‐year overall survival rates were 58.4% for IORT versus 54.9% for neoadjuvant RT alone (*p* = 0.18).

**Conclusion:**

This nationwide analysis suggests that adding IORT to treatment does not significantly improve overall survival in margin‐positive rectal cancer patients. However, due to heterogeneity in patient selection and dosing, further prospective trials are warranted to clarify its clinical role.

## Synopsis

This nationwide study analyzed 1,788 patients with margin‐positive locally advanced rectal cancer from the National Cancer Database to evaluate the impact of intraoperative radiotherapy (IORT) on overall survival in patients with pathologic T3–T4, M0 rectal adenocarcinoma and microscopically positive margins (R1). The findings indicate that adding IORT to standard treatment does not significantly improve overall survival in margin‐positive rectal cancer patients, highlighting the heterogeneity in IORT use and emphasizing the need for well‐designed prospective trials to define its clinical utility

## 1 Introduction

Rectal cancer remains a significant contributor to cancer‐related mortality, accounting for one‐third of colorectal cancer cases in the United States. Approximately 10% of patients with colorectal cancer present with locally advanced T4 disease at the time of diagnosis, posing unique therapeutic challenges [1, 2]. A multidisciplinary approach, integrating surgery, chemotherapy, and radiation therapy, has improved outcomes, with neoadjuvant chemoradiotherapy or total neoadjuvant therapy enhancing local control and reducing recurrence by downstaging tumors [3]. However, locally advanced rectal cancer (LARC) often poses challenges, particularly when achieving negative resection margins (R0) is difficult, increasing risks of local failure and mortality [4].

Intraoperative radiotherapy (IORT) is a targeted radiation therapy administered during surgery to deliver a high‐dose radiation boost to high‐risk areas while sparing surrounding healthy tissues. Beyond rectal cancer, IORT is utilized in breast, musculoskeletal, and other malignancies [5, 6, 7]. In rectal cancer, IORT serves as a valuable treatment modality for T3‐T4 tumors, recurrent disease, or cases with close or threatened margins. The European Society for Radiotherapy and Oncology (ESTRO) guidelines recommend IORT for potentially resectable T3‐T4 rectal cancers with threatened margins undergoing surgery after preoperative chemoradiotherapy, delivered as a boost to the tumor bed [8]. The National Comprehensive Cancer Network (NCCN) similarly endorses IORT as an adjunctive boost for cases with close or positive margins. Meta‐analyses have demonstrated improved local control when IORT is combined with conventional treatments, though its impact on overall survival remains debated due to the persistent risk of distant metastases [9, 10, 11].

While IORT shows promise for select cases, its utilization varies widely due to factors beyond patient and tumor characteristics, including surgeon preferences, oncologist recommendations, and institutional resources. These variations contribute to heterogeneity in clinical outcomes across treatment centers. Current evidence is limited by small sample sizes, single‐center studies, and variability in patient selection criteria. To address these gaps, this study uses a large cohort from the National Cancer Database (NCDB) to evaluate the effect of IORT on survival outcomes in rectal cancer patients with residual margin‐positive disease after surgery.

## 2 Methods

### 2.1 Study Design

This study is a retrospective cohort analysis utilizing data from the NCDB, a comprehensive oncology outcomes database that captures approximately 70% of newly diagnosed cancer cases in the United States. The NCDB provides extensive patient‐level data, including demographic characteristics, clinical and pathological features, treatment modalities, and survival outcomes, allowing for a large‐scale evaluation of IORT in rectal cancer management. The NCDB data are deidentified, and its use is exempt from institutional review board approval [12].

### 2.2 Study Population

Patients diagnosed with rectal cancer between 2006 and 2019 were identified from the NCDB. Eligible patients had pathologically confirmed T3–T4, M0 disease, received neoadjuvant chemotherapy followed by surgical resection, and had documented use of radiotherapy as part of the treatment plan, either as neoadjuvant RT and/or IORT. Only patients with microscopically positive surgical margins (R1) were included. Patients were excluded if they did not receive radiation therapy, received palliative care, did not undergo surgical resection, had macroscopic residual tumor (R2) or no residual tumor at the margin (R0), or had missing key clinical variables including margin status, treatment details, or survival outcomes.

**Figure 1:**
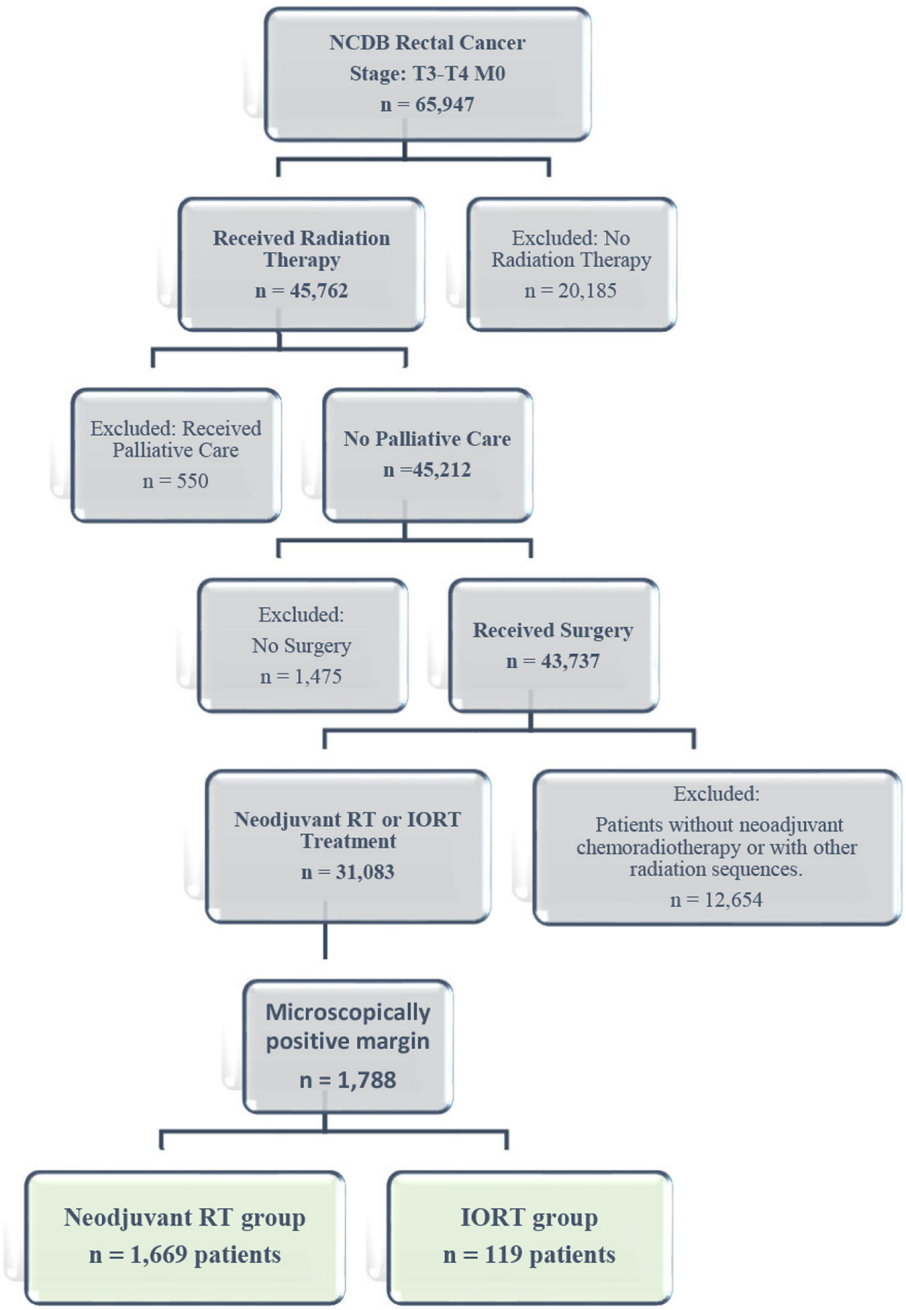
Flowchart of patient selection.

From an initial cohort of 659,747 patients diagnosed with rectal cancer in the NCDB, 20,185 patients who did not receive radiation therapy, 550 who underwent palliative care, 1475 who did not undergo surgical resection, and 12,654 patients who did not receive neoadjuvant chemotherapy or had incompatible radiation therapy sequences (such as IORT or adjuvant RT alone without neoadjuvant treatment) were excluded. After these exclusions, 31,083 patients remained. This cohort was further narrowed to 1788 patients with microscopically positive surgical margins (R1), who comprised the final study population. Based on these criteria, the final cohort was divided into two groups: those who received neoadjuvant radiotherapy (RT) alone and those treated with IORT in combination with either neoadjuvant or adjuvant RT (Figure 1).

### 2.3 Study Outcomes and Variables

Patient demographics, clinical and pathological features, and treatment‐related variables were extracted from the NCDB. Demographic and facility‐related variables included age, sex, race/ethnicity, Charlson‐Deyo comorbidity score, insurance status, facility type (e.g., academic/research program, comprehensive community cancer program), and residence location (metropolitan, urban, or rural). Clinical variables included tumor stage (pathological T3–T4, M0), receipt of neoadjuvant chemotherapy, and type of surgery (e.g., abdominoperineal resection, pelvic exenteration, partial proctosigmoidectomy). IORT was defined as intraoperative delivery of radiation therapy to the tumor bed during surgical resection using any modality (e.g., photon therapy, electron beams, or brachytherapy). Surgical margin status was pathologically defined as R1 for microscopically positive margins, indicating the presence of residual tumor cells at the resection edge.

### 2.4 Statistical Analysis

Descriptive statistics were used to summarize baseline demographic and clinical characteristics. Comparisons between the neoadjuvant RT and IORT groups were made using the Chi‐square test for categorical variables and the Wilcoxon rank‐sum test for continuous variables. To minimize selection bias and ensure a well‐balanced comparison, 1:4 propensity score matching (PSM) was performed using a nearest‐neighbor method. Matching variables included age ( ≥ 60 vs. < 60), sex, race, facility type, Charlson‐Deyo comorbidity score, insurance status, residence area, surgery type, chemotherapy regimen, and tumor stage (T and N). Covariate balance was assessed using standardized mean differences (SMD), with an SMD < 0.1 considered indicative of good balance. OS was estimated using Kaplan‐Meier analysis. Univariable and multivariable Cox proportional hazards regression analyses were performed to identify factors independently associated with survival. Hazard ratios (HR) and 95% confidence intervals (CI) were reported. All statistical analyses were conducted using R software (version 4.2.3).

## 3 Results

Among 1,788 patients with margin‐positive rectal cancer included in the analysis, 119 patients (6.7%) received intraoperative radiation therapy (IORT), while 1,669 patients (93.3%) received neoadjuvant radiation therapy (RT). As shown in Table 1, patients in the IORT group were significantly younger than those in the neoadjuvant RT group (median age: 57 vs. 60 years, *p* = 0.001). Gender distribution was similar between the groups (male: 63.9% vs. 63.5%, *p* = 0.99). Regarding insurance status, IORT patients were more likely to have private insurance (58.0% vs. 44.7%, *p* = 0.005). Facility type also differed significantly between groups (*p* < 0.001), with IORT more commonly administered at academic/research programs (75.6% vs. 30.9%) and less frequently at comprehensive community cancer programs (7.6% vs. 42.7%). Surgical approach varied as well (*p* < 0.001): IORT patients were more likely to undergo pelvic exenteration (21.8% vs. 7.7%). Multiagent chemotherapy was more frequently used in the IORT group (55.5% vs. 40.9%, *p* < 0.001).

**Table 1:** Baseline demographics and characteristics of patients.

Characteristics	Overall (*n* = 1788)	Neoadjuvant RT *N* = 1669 (93.3%)	IORT *N* = 119 (6.7%)	*p* value
Age, median (IQR)	58 (51–68)	60 (51–68)	57 (49–63)	0.001
Sex				
Male	1136 (63.5)	1060 (63.5)	76 (63.9)	0.99
Female	652 (36.5)	609 (36.5)	43 (36.1)	
Ethnicity				
African American	186 (10.4)	176 (10.5)	10 (8.4)	0.15
Caucasian	1500 (83.9)	1401 (83.9)	99 (83.2)	
Other	94 (5.3)	86 (5.2)	8 (6.7)	
Unknown	8 (0.4)	6 (0.4)	2 (1.7)	
Charlson‐Deyo score				
0	1390 (77.7)	1294 (77.5)	96 (80.7)	0.52
1	308 (17.2)	288 (17.3)	20 (16.8)	
2	55 (3.1)	54 (3.2)	1 (0.8)	
3 or more	35 (2.0)	33 (2.0)	2 (1.7)	
Insurance				
Medicaid	216 (12.1)	198 (11.9)	18 (15.1)	0.005
Medicare	614 (34.3)	591 (35.3)	23 (19.3)	
Not Insured/Self‐pay	100 (5.6)	94 (5.6)	6 (5.0)	
Other government	21 (1.2)	21 (1.3)	0 (0)	
Private Insurance	815 (45.6)	746 (44.7)	69 (58.0)	
Unknown	22 (1.2)	19 (1.1)	3 (2.5)	
Facility Type				
Community Cancer Program	88 (4.9)	83 (5.0)	5 (4.2)	< 0.001
Comprehensive Community Cancer Program	722 (40.4)	713 (42.7)	9 (7.6)	
Academic/Research Program	605 (33.8)	515 (30.9)	90 (75.6)	
Integrated Network Cancer Program	373 (20.9)	358 (21.4)	15 (12.6)	
Residence Area				
Metropolitian	1482 (82.9)	1392 (83.4)	90 (75.7)	0.09
Rural	38 (2.1)	35 (2.1)	3 (2.5)	
Urban	268 (15.0)	242 (14.5)	26 (21.8)	
Surgery Type				
Partial proctosigmoidectomy	816 (45.6)	779 (46.7)	37 (31.1)	< 0.001
Abdominoperineal resection	654 (36.6)	613 (36.7)	41 (34.5)	
Pelvic exenteration	155 (8.7)	129 (7.7)	26 (21.8)	
Pull through with sphincter preservation	98 (5.5)	89 (5.3)	9 (7.6)	
Total proctocolectomy	38 (2.1)	36 (2.2)	2 (1.7)	
Surgery, NOS	17 (1.0)	14 (0.8)	3 (2.5)	
Proctectomy, NOS	10 (0.6)	9 (0.5)	1 (0.8)	
Chemotherapy				
Single‐agent	880 (49.2)	827 (49.6)	53 (44.5)	< 0.001
Multiagent	749 (41.9)	683 (40.9)	66 (55.5)	
Not documented^a^	159 (8.9)	159 (9.5)	0 (0)	
T Stage				
T3	1263 (70.6)	1165 (69.8)	98 (82.4)	0.005
T4	525 (29.4)	504 (30.2)	21 (17.6)	
N Stage				
N0	763 (42.7)	713 (42.7)	50 (42.0)	< 0.001
N1	633 (35.4)	575 (34.5)	58 (48.7)	
N2	392 (21.9)	381 (22.8)	11 (9.2)	

The data were expressed as frequencies and percentages (%) or median and interquartile range (IQR).

IORT, Intraoperative radiation therapy; RT, Radiation therapy; NOS, Not otherwise specified.

^a^ Chemotherapy administered as first course therapy, but the type and number of agents is not documented in patient record.

**Figure 2:**
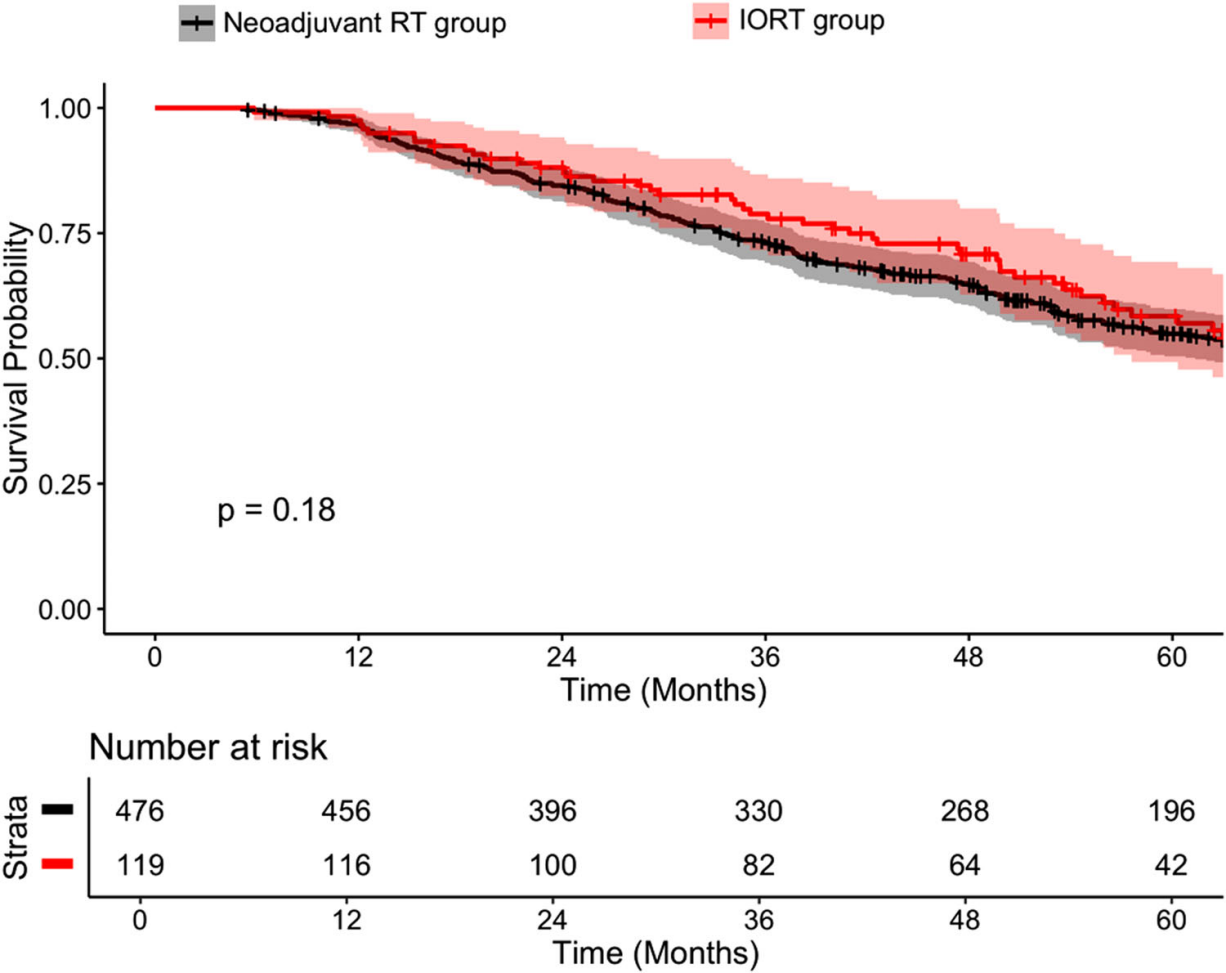
Kaplan‐meier survival curve comparing treatment approach in a matched cohort. *(5‐Year Overall Survival: Neoadjuvant RT: 54.9%, IORT 58.4%; p* = *0.18)*.

After propensity score matching, 119 IORT patients were compared with 476 neoadjuvant RT patients (Supplemental Table 1). In the matched cohort, 5‐year overall survival (OS) rates were 58.4% for IORT versus 54.9% for neoadjuvant RT (*p* = 0.18; Figure 2). In contrast, the unmatched cohort showed a more pronounced survival difference (IORT: 64.1% vs. neoadjuvant RT: 58.4%, *p* = 0.0013; Supplemental Figure 1). In univariable analysis (Table 2), IORT was associated with significantly lower mortality compared to neoadjuvant RT (HR: 0.63, 95% CI: 0.47–0.83, *p* < 0.001). However, in multivariable analysis adjusting for confounding factors, IORT was not independently associated with improved OS (HR: 0.76, 95% CI: 0.57–1.02, *p* = 0.07).

**Table 2:** Univariable and multivariate cox regression analysis of factors associated with survival.

Variables	Univariable analysis			Multivariable analysis		
	HR	95% CI	*p* value	HR	95% CI	*p* value
Treatment (ref = neoadjuvant RT)						
IORT	0.63	0.47–0.83	< 0.001	0.76	0.57–1.02	0.07
Age (ref = < 60)						
≥ 60 years	1.47	1.31–1.66	< 0.001	1.25	1.01–1.47	0.005
Sex (ref = Female)						
Male	0.99	0.87–1.12	0.83	1.02	0.89–1.17	0.76
Ethnicity (ref = African American)						
Caucasian	0.87	0.72–1.06	0.16	0.8	0.65–0.98	0.03
Other	0.98	0.71–1.36	0.92	1.00	0.72–1.4	0.98
Unknown	0.65	0.24–1.77	0.39	0.68	0.68–1.87	0.46
Charlson‐Deyo score (ref = 0)						
1	1.07	0.91–1.26	0.42	0.98	0.83–1.16	0.8
2	1.98	1.44–2.73	< 0.001	1.68	1.21–2.35	0.002
3 or more	1.53	0.99–2.37	0.05	1.2	0.77–1.87	0.41
Insurance (ref = Medicaid)						
Medicare	1.48	1.2–1.83	< 0.001	1.32	1.04–1.67	0.02
Not Insured/Self‐pay	1.04	0.76–1.43	0.81	1.0	0.72–1.37	0.99
Other Government	1.17	0.66–2.08	0.58	0.97	0.54–1.75	0.92
Private Insurance	0.9	0.73–1.11	0.32	0.91	0.74–1.13	0.41
Unknown	0.99	0.52–1.89	0.99	0.98	0.53–1.94	0.98
Facility Type (ref = Community Cancer Program)						
Comprehensive Community Cancer Program	1.0	0.76–1.33	0.98	1.15	0.86–1.54	0.33
Academic/Research Program	0.84	0.63–1.12	0.25	1	0.74–1.34	0.98
Integrated Network Cancer Program	0.95	0.71–1.13	0.73	1.06	0.79–1.43	0.69
Location (ref = Metropolitian)						
Rural	0.9	0.59–1.37	0.62	0.98	0.64–1.49	0.92
Urban	1.04	0.88–1.24	0.64	1.02	0.85–1.21	0.86
Surgery Type (ref = Surgery, NOS)						
Pull through with sphincter preservation	1.15	0.56–2.33	0.71	1.17	0.57–2.41	0.67
Total proctocolectomy, NOS	1.64	0.76–3.54	0.21	1.57	0.72–3.42	0.26
Abdominoperineal resection	1.44	0.75–2.79	0.28	1.35	0.69–2.65	0.38
Partial proctosigmoidectomy	1.2	0.63–2.33	0.58	1.15	0.59–2.24	0.69
Pelvic exenteration	1.64	0.83–3.26	0.16	1.53	0.77–3.08	0.23
Proctectomy, NOS	0.57	0.16–2.12	0.4	0.46	0.12–1.71	0.24
Chemotherapy (ref = Single‐agent)						
Multiagent	0.75	0.66–0.85	< 0.001	0.78	0.69–0.89	< 0.001
T Stage (ref = T3)						
T4	1.47	1.29–1.68	< 0.001	1.5	1.31–1.72	< 0.001
N Stage (ref = N0)						
N1	1.29	1.11–1.49	0.003	1.41	1.21–1.63	< 0.001
N2	1.74	1.49–2.04	0.002	1.99	1.7–2.34	< 0.001

The data were expressed as frequencies and percentages (%) or median and interquartile range (IQR).

CI, confidence interval; HZ, Hazard Ratio; IORT, intraoperative radiation therapy; NOS, not otherwise specified; RT, radiation therapy.

Several variables were independently associated with survival outcomes. Age ≥ 60 was associated with worse survival (HR: 1.25, *p* = 0.005). Race was significant, with Caucasian patients demonstrating better survival than African American patients (HR: 0.80, 95% CI: 0.65–0.98, *p* = 0.03). A Charlson‐Deyo score of 2 was associated with higher mortality compared to a score of 1 (HR: 1.68, *p* = 0.002). Medicare coverage was linked to poorer outcomes relative to Medicaid (HR: 1.32, *p* = 0.02). Use of multiagent chemotherapy showed a survival benefit over single‐agent therapy (HR: 0.78, 95% CI: 0.69–0.89, *p* < 0.001). Advanced tumor features were also linked to worse outcomes, including T4 stage (HR: 1.50, *p* < 0.001), N1 status (HR: 1.41, *p* < 0.001), and N2 status (HR: 1.99, *p* < 0.001).

## 4 Discussion

This study, utilizing a large cohort from the NCDB, demonstrates that IORT combined with adjuvant or neoadjuvant radiation therapy offers no clear survival advantage compared to neoadjuvant RT alone in patients with margin‐positive, locally advanced T3–T4 rectal cancer. Despite its targeted therapeutic approach, IORT was predominantly administered at academic and research centers and was more frequently used in younger patients undergoing complex surgical procedures, such as pelvic exenteration—suggesting selective application in high‐risk clinical scenarios. These findings indicate that while IORT may be reserved for the most challenging cases, its overall survival benefit appears limited. This is consistent with previous meta‐analyses, which suggest that IORT may enhance local control but does not clearly translate into long‐term survival improvement [13, 14].

Over the past two decades, the long‐term impact of IORT on oncologic outcomes has remained unclear due to the lack of standardization in treatment protocols, patient selection criteria, and outcome reporting. The heterogeneity of patient populations and treatment regimens in the literature complicates direct comparisons, even within meta‐analyses, limiting the ability to draw definitive conclusions. Most available studies are small, single‐institution series with significant variability in methodology, further contributing to inconsistent findings [9, 13, 14, 15]. A systematic review by Wiig et al. [13] in 2014 analyzed 18 studies on LARC, including 13 that incorporated IORT and five that did not. The analysis found no OS benefit with IORT, even among patients who achieved R0 resections. While local recurrence rates were lower in patients with R1 or R2 resections who received IORT (33% vs. 82%), there was no significant difference in OS. Furthermore, the only randomized controlled trial included in the review failed to demonstrate a survival or local control benefit with IORT. The authors concluded that existing data are inconsistent, requiring cautious interpretation, and that IORT is unlikely to improve OS, with only a potential benefit in reducing LR in patients with non‐R0 resections. These findings are further supported by meta‐analyses from Liu et al. [14] and Fahy et al. [11], which reinforce that although IORT can enhance local control when added to standard multimodal therapy, it does not significantly impact long‐term survival.

The 2023 NCCN Rectal Cancer Guidelines [10] recommend that IORT, if available, should be considered for very close or positive margins after resection, particularly in patients with T4 disease following total neoadjuvant therapy. Additionally, the ESTRO‐ACROP guidelines [8], based on a review of 2,843 patients across 21 studies, advocate for IORT in potentially resectable T3–T4 rectal cancers after preoperative chemoradiotherapy. They emphasize that the decision to administer IORT should be based on intraoperative findings, margin status, and multidisciplinary collaboration between the surgeon and radiation oncologist. According to these recommendations, IORT is most appropriate for cases with gross residual disease, microscopically positive margins, or close radial soft tissue margins ( < 2–5 mm), where additional local control measures are warranted.

In clinical practice, IORT administration decisions integrate surgical findings, margin status evaluations, pretreatment physical assessments, and comprehensive imaging studies. This complex determination typically represents a collaborative intraoperative judgment between the surgeon and radiation oncologist. While some centers rely on preoperative imaging to identify high‐risk cases, others utilize intraoperative frozen sections to guide real‐time decision‐making. When surgical margins are found to be threatened or positive, and further resection is not feasible, IORT may be employed as an adjunct to improve local control. Given this treatment paradigm, our study specifically focused on margin‐positive T3‐T4 rectal cancer patients, as recommended by clinical guidelines, to evaluate whether IORT impacts overall survival in this high‐risk group. Our nationwide study found no survival advantage with IORT. However, its potential to reduce local recurrence remains an important consideration. Several studies have demonstrated that IORT significantly lowers local recurrence rates in patients with microscopically involved circumferential resection margins after surgery for locally advanced rectal cancer. Consequently, IORT continues to be an integral part of the treatment strategy for these high‐risk patients to minimize local re‐recurrence [16, 17, 18]. Contrasting these findings, a French randomized study did not show a significant improvement in local control or disease‐free survival with the addition of IORT to preoperative radiation therapy [19].

IORT offers a targeted radiation approach that minimizes toxicity to surrounding organs such as the bladder, ureter, prostate, vagina, and uterus while also protecting the small bowel, a critical dose‐limiting structure in external beam radiotherapy (EBRT). Conventional long‐course neoadjuvant EBRT is typically restricted to doses at or below 50‐54 Gy due to the risk of complications, including ulceration and strictures [20]. In contrast, IORT enables dose escalation to biologically effective levels two to three times higher than what can be safely delivered with EBRT, potentially improving local tumor control without increasing systemic toxicity. In terms of safety, studies have demonstrated that IORT is a well‐tolerated treatment option for patients with locally advanced or recurrent rectal cancer. A comparison of patients undergoing rectal resection with or without IORT found no significant differences in major postoperative complications, including anastomotic leakage, hospital stay, or wound infection rates [14, 21]. However, IORT was associated with longer operative times but did not appear to prolong hospitalization [22]. While overall complication rates remain comparable between groups, some studies have reported an increased incidence of wound‐related complications, particularly infections and pelvic abscesses, occurring in approximately 25% of IORT patients [15, 23].

This study has several limitations inherent to its retrospective design. First, our analysis was restricted to OS as the primary endpoint, as disease‐free survival and local control rates were not recorded in the NCDB database. This is a critical limitation given that IORT is primarily utilized to enhance local tumor control. Second, essential details, including IORT dose, radiation techniques, and anatomical relationships, were absent, limiting a comprehensive analysis of their effects. Third, NCDB variable definitions prevented us from distinguishing whether IORT was paired with neoadjuvant or adjuvant radiotherapy. The data set combines IORT with radiation administered before or after surgery without clarifying the sequence, reducing the precision of our treatment group definitions and potentially introducing clinical heterogeneity. These factors, known to influence survival in IORT patients, may obscure variations in treatment efficacy. Additionally, the NCDB does not differentiate between primary and recurrent rectal cancer cases, which is a crucial factor in assessing IORT′s effectiveness. Despite these limitations, the study has significant strengths. The use of a large, nationally representative cohort allowed for adequate power to assess IORT utilization and outcomes. Propensity score matching was employed to reduce confounding and enhance the validity of our comparisons. While IORT has been in use for over three decades, much of the existing literature is based on small, single‐institution studies with limited generalizability. Even meta‐analyses are challenged by variations in patient populations, treatment protocols, and outcome reporting. This study adds valuable nationwide data to the ongoing effort to clarify the role of IORT in rectal cancer management.

## 5 Conclusion

In this large nationwide analysis of patients with margin‐positive locally advanced rectal cancer, the addition of IORT to standard treatment did not significantly improve overall survival. However, given the heterogeneity in patient selection and treatment practices, well‐designed prospective trials are needed to clarify its clinical utility and optimize patient selection.

## Conflicts of Interest

Dr. Emre Gorgun is a consultant for Boston Scientific, Intuitive, and DiLumen. The other authors declare no conflicts of interest.

## Supporting information


**Supplemental 1:** Kaplan‐meier survival curve comparing treatment approach in a unmatched cohort

**Supplemental 1:** Baseline demographics and characteristics of patients (Matched Cohort)

## Data Availability

The data used in this study were derived from a deidentified file provided by the National Cancer Database (NCDB), a joint project of the Commission on Cancer of the American College of Surgeons and the American Cancer Society. The American College of Surgeons and the Commission on Cancer have not verified and are not responsible for the analytic methods or conclusions drawn by the investigators. The NCDB data set contains no patient‐level identifiers and is publicly available upon request and approval via the American College of Surgeons website: https://www.facs.org/quality-programs/cancer-programs/national-cancer-database/.
