# Supplementary material for: The Impact of Intraoperative Radiotherapy on Margin Positive Locally Advanced Rectal Cancer: A Propensity‐Matched Analysis of The National Cancer Database
Source: J Surg Oncol. 2025 Sep 26;132(7):1257–65. doi: 10.1002/jso.70102 (PMC12579357; doi:10.1002/jso.70102)
Supplement: Supplementary file 1 — Supplemental 1: Kaplan‐meier survival curve comparing treatment approach in a unmatched cohort [file JSO-132-1257-s002.docx]

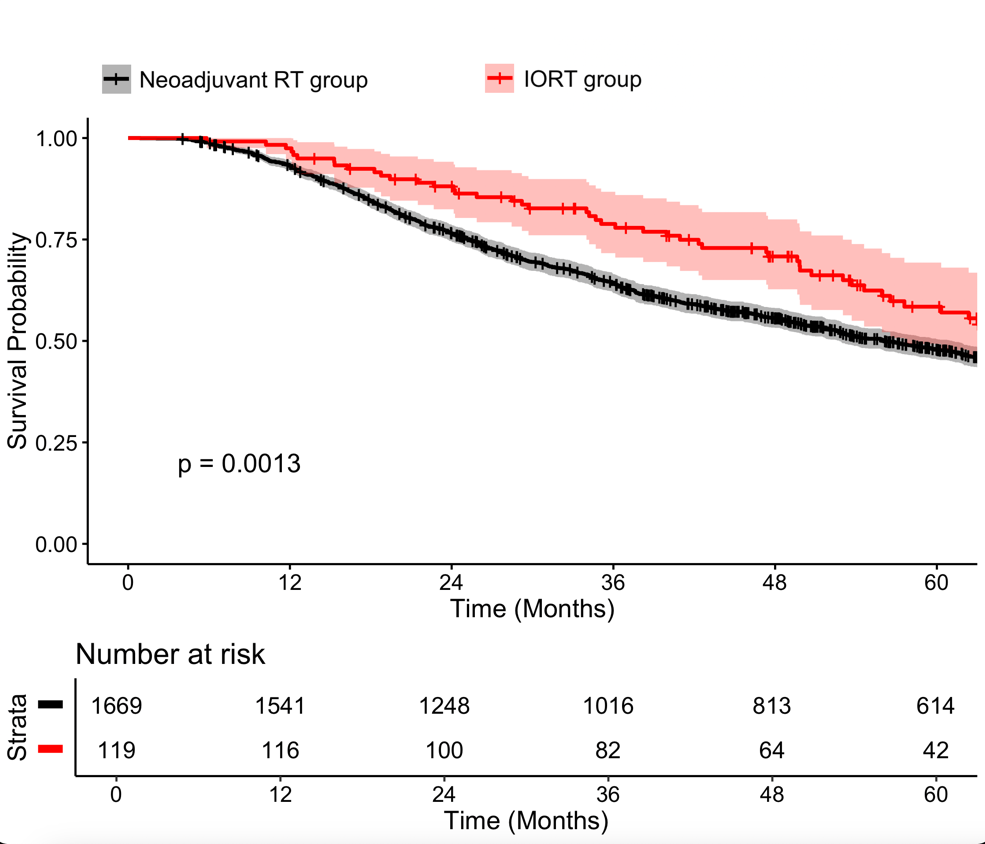


| **Supplemental 1** Kaplan-meier survival curve comparing treatment approach in a unmatched cohort  *(5-Year Overall Survival:* *Neoadjuvant RT: 58.4%, IORT 64.1% ; p = 0.0013)* |
| --- |
