# Supplementary material for: The Impact of Intraoperative Radiotherapy on Margin Positive Locally Advanced Rectal Cancer: A Propensity‐Matched Analysis of The National Cancer Database
Source: J Surg Oncol. 2025 Sep 26;132(7):1257–65. doi: 10.1002/jso.70102 (PMC12579357; doi:10.1002/jso.70102)
Supplement: Supplementary file 2 — Supplemental 1: Baseline demographics and characteristics of patients (Matched Cohort) [file JSO-132-1257-s001.docx]

| **Supplemental 1** Baseline demographics and characteristics of patients (Matched Cohort) | | | | | |
| --- | --- | --- | --- | --- | --- |
| **Characteristics** |  | Overall (n=595) | Neoadjuvant RT  N = 476 | IORT  N = 119 | p-value |
| Age, median (IQR) |  | 57 (50 - 64) | 57 (49 - 63) | 57 (51 - 64) | 0.85 |
| Sex |  |  |  |  |  |
| Male |  | 373 (62.7) | 297 (63.9) | 76 (62.4) | 0.77 |
| Female |  | 222 (37.3) | 182 (36.1) | 43 (37.6) |  |
| Ethnicity |  |  |  |  |  |
| African-American |  | 54 (9.1) | 44 (9.2) | 10 (8.4) | 0.77 |
| Caucasian |  | 502 (84.4) | 403 (84.7) | 99 (83.2) |  |
| Other |  | 33 (5.5) | 25 (5.3) | 8 (6.7) |  |
| Unknown |  | 6 (1.0) | 4 (0.8) | 2 (1.7) |  |
| Charlson-Deyo score |  |  |  |  |  |
| 0 |  | 478 (80.3) | 382 (80.3) | 96 (80.7) | 1 |
| 1 |  | 102 (17.1) | 82 (17.2) | 20 (16.8) |  |
| 2 |  | 5 (0.8) | 4 (0.8) | 1 (0.8) |  |
| 3 or more |  | 10 (1.7) | 8 (1.7) | 2 (1.7) |  |
| Insurance |  |  |  |  |  |
| Medicaid |  | 88 (14.8) | 70 (14.7) | 18 (15.1) | 0.95 |
| Medicare |  | 130 (21.8) | 107 (22.5) | 23 (19.3) |  |
| Not Insured/Self-pay |  | 30 (5.0) | 24 (5.0) | 6 (5.0) |  |
| Other government |  | 0 (0) | 0 (0) | 0 (0) |  |
| Private Insurance |  | 335 (56.3) | 266 (55.9) | 69 (58.0) |  |
| Unknown |  | 12 (2.0) | 9 (1.9) | 3 (2.5) |  |
| Facility Type |  |  |  |  |  |
| Community Cancer Program |  | 28 (4.7) | 23 (4.8) | 5 (4.2) | 0.66 |
| Comprehensive Community Cancer Program |  | 48 (8.1) | 39 (8.2) | 9 (7.6) |  |
| Academic/Research Program |  | 424 (71.3) | 334 (70.2) | 90 (75.6) |  |
| Integrated Network Cancer Program |  | 95 (16.0) | 80 (16.8) | 15 (12.6) |  |
| Residence Area |  |  |  |  |  |
| Metropolitian |  | 481 (80.8) | 391 (82.1) | 90 (75.7) | 0.27 |
| Rural |  | 11 (1.8) | 8 (1.7) | 3 (2.5) |  |
| Urban |  | 103 (17.3) | 77 (16.2) | 26 (21.8) |  |
| Surgery Type |  |  |  |  |  |
| Partial proctosigmoidectomy |  | 207 (34.8) | 170 (35.7) | 37 (31.1) | 0.34 |
| Abdominoperineal resection |  | 229 (38.5) | 188 (39.5) | 41 (34.5) |  |
| Pelvic exenteration |  | 89 (15.0) | 63 (12.3) | 26 (21.8) |  |
| Pull through with sphincter preservation |  | 43 (7.2) | 34 (7.1) | 9 (7.6) |  |
| Total proctocolectomy |  | 13 (2.2) | 11 (2.3) | 2 (1.7) |  |
| Surgery, NOS |  | 11 (1.8) | 8 (1.7) | 3 (2.5) |  |
| Proctectomy, NOS |  | 3 (0.5) | 2 (0.4) | 1 (0.8) |  |
| Chemotherapy |  |  |  |  |  |
| Single-agent |  | 278 (46.7) | 225 (47.3) | 53 (44.5) | 0.66 |
| Multi-agent |  | 317 (53.3) | 251 (52.7) | 66 (55.5) |  |
| Not documented^a^ |  | 0 (0) | 0 (0) | 0 (0) |  |
| T Stage |  |  |  |  |  |
| T3 |  | 469 (78.8) | 371 (77.9) | 98 (82.4) | 0.35 |
| T4 |  | 126 (21.2) | 105 (22.1) | 21 (17.6) |  |
| N Stage |  |  |  |  |  |
| N0 |  | 280 (47.1) | 230 (48.3) | 50 (42.0) | 0.23 |
| N1 |  | 249 (41.8) | 191 (40.1) | 58 (48.7) |  |
| N2 |  | 66 (11.1) | 55 (11.6) | 11 ( 9.2) |  |
| The data were expressed as frequencies and percentages (%) or median and interquartile range (IQR)  ^a^Chemotherapy administered as first course therapy, but the type and number of agents is not documented in patient record.  IORT: Intraoperative radiation therapy, RT: Radiation therapy, NOS: Not otherwise specified. | | | | | |
